# Dietary *Hypsizygus marmoreus* Stipe May Improve Survival of Juvenile Largemouth Bass (*Micropterus salmoides*) Through Metabolic Regulation

**DOI:** 10.3390/ani16142166

**Published:** 2026-07-13

**Authors:** Ershu Lin, Kejia Weng, Qingyu Huang, Ying Zhou, Yuchen Zhuo

**Affiliations:** 1 Fujian Freshwater Fisheries Research Institute, Fuzhou 350000, China; ershulin123@163.com (E.L.); boicexx@gmail.com (K.W.); 2 College of Life Science, Fujian Agriculture and Forestry University, Fuzhou 350000, China; hqy18402801643@163.com (Q.H.); yingzhou1003@163.com (Y.Z.); 3 College of Animal Science, Fujian Agriculture and Forestry University, Fuzhou 350000, China

**Keywords:** *Hypsizygus marmoreus* stipe, largemouth bass, survival rate, metabolic regulation, functional feed additive

## Abstract

The stipe of *Hypsizygus marmoreus* is a low-value byproduct generated during mushroom processing. Reutilization of this agricultural waste as a functional feed additive may provide both economic and environmental benefits for sustainable aquaculture. This study evaluated the effects of dietary *H. marmoreus* stipe (HMS) supplementation on the growth performance and survival of juvenile Micropterus salmoides and explored the underlying metabolic and transcriptional responses. Juvenile largemouth bass (initial body weight: 1.13 ± 0.05 g) were fed diets containing 0% (HMS0), 5% (HMS5), or 10% (HMS10) HMS for 46 days. HMS supplementation did not significantly affect growth performance but significantly increased survival rate in both HMS5 and HMS10 groups. Widely targeted metabolomic profiling suggested that dietary HMS may drive prominent shifts in the hepatic and gastric metabolic profiles, with potential changes observed in pathways associated with pyrimidine metabolism, glycerophospholipid metabolism, and ABC transporters. Hepatic transcriptomic analysis further identified differentially expressed genes enriched in these pathways. Integrated multi-omics analysis indicated potential coordinated associations between key metabolites, including orotic acid, ureidosuccinic acid, LPG (16:1), and Val-Cys, and genes involved in nucleotide metabolism, lipid metabolism, and transporter functions, such as *DPYD* (dihydropyrimidine dehydrogenase), *NDK* (nucleoside diphosphate kinase), *ENTP5* (ectonucleoside triphosphate diphosphohydrolase 5), *NT5D2* (5′-nucleotidase domain containing 2), *GPT3L* (glycerol-3-phosphate acyltransferase-like), *ABCA1* (ATP-binding cassette subfamily A member 1), and *ABCD4* (ATP-binding cassette subfamily D member 4). In addition, antioxidant enzyme activities were significantly enhanced in the HMS5 group, whereas no further improvement was observed at the 10% supplementation level. Collectively, these findings suggest that dietary HMS supplementation may enhance the survival of largemouth bass via coordinated metabolic regulation and antioxidant responses. This study highlights the potential application of mushroom-processing byproducts as sustainable functional feed ingredients in aquaculture.

## Simple Summary

Large-scale mushroom production generates substantial amounts of agricultural waste, including the stipe (stem base) of *Hypsizygus marmoreus*, which is often discarded despite its rich nutritional composition. In this study, we evaluated whether dietary supplementation with *H. marmoreus* stipe could improve the health and survival of juvenile *Micropterus salmoides*, an economically important aquaculture species. Our results showed that dietary supplementation with 5% or 10% mushroom stipe significantly increased fish survival without affecting growth performance. Multi-omics analyses further suggested that dietary supplementation with mushroom stipe may induce changes in metabolic pathways linked to nucleotide metabolism, membrane lipid metabolism, and antioxidant regulation. In addition, moderate supplementation (5%) enhanced antioxidant enzyme activities. These findings suggest that *H. marmoreus* stipe has potential as a sustainable functional feed ingredient in aquaculture and provides a promising strategy for recycling mushroom industry byproducts while improving fish health and survival.

## 1. Introduction

Largemouth bass (*Micropterus salmoides*) is an economically important freshwater fish species widely cultivated in China due to its fast growth, delicious flesh, and high market value [1,2]. According to China Fishery Statistical Yearbook 2023, the annual output of largemouth bass in China in 2022 was 802,500 tons, with a production value exceeding 20 billion yuan [3]. The global total aquaculture production of largemouth bass stood at approximately 805,000 tons that year, with China accounting for more than 99.5% of the worldwide supply. With the rapid expansion of intensive aquaculture practices, largemouth bass farming frequently faces challenges associated with high stocking density, deteriorating water quality, oxidative stress, and increased susceptibility to infectious diseases, resulting in substantial economic losses. High stocking density in intensive systems accelerates horizontal transmission of lethal viral, bacterial and parasitic pathogens, which cause large-scale, abrupt mortality outbreaks across commercial farms [4,5,6,7]. Therefore, developing effective and sustainable nutritional strategies to improve fish health and survival has become a major research priority in aquaculture.

Dietary supplementation with functional feed additives, such as prebiotics [8] and plant extracts [9], has emerged as a promising approach to enhance immune function and stress tolerance in fish. Recent studies have demonstrated that dietary components capable of modulating nucleotide metabolism can play important roles in immune regulation and stress adaptation. Nucleotides can be fully biosynthesized endogenously by largemouth bass under low-stress culture conditions and are classified as non-essential nutrients to meet basic metabolism. Yet during rapid juvenile growth, intensive environmental stress, or pathogen exposure, endogenous nucleotide synthesis fails to support optimal growth, lymphocyte proliferation, macrophage activation, immunoglobulin synthesis and tissue repair, making nucleotides semi-essential that necessitate dietary supplementation. Edible macrofungi are recognized as natural reservoirs of abundant RNA and free nucleoside/nucleotide compounds, with considerable pyrimidine pools accumulated in their stipe tissues, making mushroom agricultural waste a promising raw material for exogenous nucleotide supply in aquafeed formulations [10,11]. In aquaculture species, dietary nucleotide supplementation has been reported to improve intestinal morphology, enhance stress tolerance, and strengthen both innate and adaptive immune responses [12]. For example, Chen et al. reported that dietary nucleotide supplementation (0.4–0.8 g kg^−1^) promoted growth performance, enhanced immunity, and improved intestinal morphology and disease resistance against *Aeromonas hydrophila* in juvenile largemouth bass [13].

Edible mushrooms are rich in bioactive compounds including polysaccharides, dietary fibers, ergosterol, and phenolic compounds, which possess immunomodulatory, antioxidant, and antimicrobial properties. *Hypsizygus marmoreus* (also known as beech mushroom or bunashimeji) is a widely cultivated edible mushroom [14,15]. During industrial processing, the basal stipe of *H. marmoreus*, accounting for approximately 20–30% of the fruiting body weight, is commonly discarded as agricultural waste despite its considerable nutritional value. This byproduct contains substantial levels of crude protein, dietary fiber, and mineral components, suggesting its potential as a low-cost functional feed ingredient. However, the effects of dietary H. marmoreus stipe (HMS) supplementation on fish health and survival, as well as the underlying molecular mechanisms, remain largely unexplored.

To date, only limited studies have investigated the application of mushroom byproducts in aquafeeds. For example, dietary supplementation with spent mushroom substrate derived from Cordyceps militaris significantly enhanced skin mucus lysozyme activity, serum immune parameters, and resistance against Streptococcus agalactiae infection in Nile tilapia [16]. In addition, co-fermented protein produced from king oyster mushroom root waste and soybean meal has been shown to improve growth performance and immune responses in juvenile largemouth bass [17]. Nevertheless, most previous studies have focused on fermented products or crude extracts, whereas the direct utilization of unprocessed mushroom stipe byproducts remains rarely investigated. More importantly, the molecular mechanisms by which mushroom-derived dietary components regulate fish physiology and survival have not been systematically elucidated using integrated multi-omics approaches.

In this study, we hypothesized that dietary HMS supplementation could improve the physiological health and survival of largemouth bass through coordinated metabolic regulation. To test this hypothesis, juvenile largemouth bass were fed diets containing 0%, 5%, or 10% HMS for 46 days. Growth performance and survival rate were evaluated, while widely targeted metabolomic profiling of liver and stomach tissues, hepatic transcriptomic analysis, and antioxidant enzyme activity assays were conducted to investigate the underlying molecular responses. By integrating metabolomic and transcriptomic datasets, we aimed to identify key metabolic pathways associated with the beneficial effects of HMS supplementation on fish survival. The findings of this study provide a scientific basis for the reutilization of mushroom-processing byproducts as sustainable functional feed ingredients in aquaculture.

## 2. Materials and Methods

### 2.1. Ethical Statement

All animal experiments were approved by the Animal Care and Use Committee of Fujian Freshwater Fisheries Research Institute (Fuzhou, China), (ID Number: FFRIF-DW-2024-10; approval date: 11 March 2024) and were performed in accordance with the Guide for the Care and Use of Laboratory Animals.

### 2.2. Diet Preparation

The basal stipe of *H. marmoreus* was obtained from Gutian Edible Fungi Research Institute (Ningde, China). The chemical composition of the stipe powder was as follows: crude protein, 18%; crude lipid, 2%; crude fiber, 20%; and ash, 12%. The dried stipe material was ground to pass through a 320-μm sieve and incorporated into a commercial basal diet (Fujian Tianma Technology Group Co., Ltd., Fuqing, China) at supplementation levels of 0% (HMS0), 5% (HMS5), and 10% (HMS10) (Table 1). HMS was used to partially replace multiple basal diet ingredients (fish meal, flour, soybean meal, yeast, etc.) in an equal mass manner. After thorough mixing, the diets were processed into 2-mm pellets using a laboratory twin-screw extruder (Valva-60, Guangzhou Valva Machinery Equipment Co., Ltd., Guangzhou, China) at 110 °C and 1.83 MPa. The pellets were dried at 55 °C and stored at −20 °C until use.

### 2.3. Fish Husbandry and Experimental Design

The feeding trial was conducted at Fujian Freshwater Fisheries Research Institute. A total of 270 juvenile *Micropterus salmoides* (initial body weight: 1.13 ± 0.05 g) were acclimated for one week using a commercial diet (Fujian Tianma Technology Group Co., Ltd., China) and fed to apparent satiation twice daily (07:00 and 19:00). Following acclimation, fish were randomly assigned to three dietary treatment groups (HMS0, HMS5, and HMS10), with three replicate tanks per treatment and 30 fish per tank.

Fish were fed their respective diets to apparent satiation twice daily for 46 days. Uneaten feed and feces were removed by siphoning prior to each feeding. A natural photoperiod was maintained throughout the experiment, and continuous aeration was supplied using an air pump to maintain dissolved oxygen levels above 5 mg L^−1^. Water quality parameters were monitored daily and maintained as follows: temperature, 27–30 °C; pH, 7.6–7.9; ammonia nitrogen, ≤0.1 mg L^−1^; and nitrite, ≤0.01 mg L^−1^.

### 2.4. Sample Collection

At the end of the feeding trial, fish were fasted for 24 h and anesthetized with tricaine methanesulfonate (MS-222, 100 mg L^−1^). The total number of surviving fish in each tank was recorded to calculate survival rate, and all fish were weighed individually to determine final body weight. Four fish were sampled from each tank, and the tissues of the 4 fish in one tank were homogenized and pooled into one mixed sample. Three replicate tanks per group finally formed 3 biological replicates (*n* = 3) for subsequent metabolomic, transcriptomic, and biochemical analyses.

### 2.5. Growth Performance and Survival Rate

Body weight and body length were measured at the beginning and end of the experiment. Survival rate was calculated using the following formula: (final number of fish/initial number of fish) × 100%.

Feed conversion ratio (FCR), specific growth rate (SGR), and condition factor (CF) were calculated as follows

$$FCR=FI/(W_{t}-W_{0});$$

$$SGR(\%/day)=100\times (\ln W_{t}-\ln W_{0})/t;$$

$$CF=100\times W/L^{3};$$

where FI is total feed intake; $W_{0}$ and $W_{t}$ are the initial and final average body weight (g); t is the feeding trial duration (days); W is individual body weight (g), and L is body length (cm).

### 2.6. Widely Targeted Metabolomic Analysis

Widely targeted metabolomic profiling was performed on liver and stomach samples (*n* = 3 per group) by Metware Biotechnology Co., Ltd. (Wuhan, China). Sample extraction and UPLC-MS/MS analyses were conducted according to the company’s standard protocols. Briefly, approximately 20 mg of tissue was homogenized and extracted using 70% methanol containing internal standards. Following centrifugation, the supernatant was analyzed using an ExionLC AD UPLC system coupled with a QTRAP 6500+ mass spectrometer (SCIEX, Marlborough, MA, USA).

Metabolites were identified based on retention time, precursor ions, and fragment ion spectra using the Metware database (MWDB). Principal component analysis (PCA), orthogonal partial least squares-discriminant analysis (OPLS-DA), and differential metabolite screening were performed. Differential metabolites were identified using the criteria of variable importance in projection (VIP) > 1 and *p* < 0.05 (Student’s *t*-test). *p*-values were adjusted via a standard FDR correction procedure (Benjamini–Hochberg method). KEGG pathway enrichment analysis was conducted using a hypergeometric test.

### 2.7. De Novo Transcriptomic Analysis

Total RNA was extracted from liver tissues (*n* = 3 per group) using TRIzol reagent (Invitrogen, Carlsbad, CA, USA) according to the manufacturer’s instructions. RNA quality and integrity were evaluated by agarose gel electrophoresis and a Qsep400 Bioanalyzer (NIPPON Genetics EUROPE, Düren, Germany). cDNA libraries were constructed using the Illumina TruSeq Stranded mRNA Library Preparation Kit and sequenced on the Illumina NovaSeq 6000 platform with paired-end 150 bp reads.

Raw reads were filtered using fastp to remove adapter sequences and low-quality reads. De novo transcriptome assembly was conducted using Trinity v2.13.2. Functional annotation of unigenes was performed against the NR, Swiss-Prot, KOG, GO, and KEGG databases using DIAMOND. Gene expression levels were quantified as fragments per kilobase of transcript per million mapped reads (FPKM) using RSEM. Differentially expressed genes (DEGs) were identified using DESeq2 with thresholds of |log_2_FC| ≥ 1 and false discovery rate (FDR) < 0.05. GO and KEGG enrichment analyses were performed using clusterProfiler 4.21.1.

### 2.8. Integrated Metabolomic and Transcriptomic Analysis

To identify pathways coordinately regulated at both metabolite and transcript levels, KEGG enrichment results obtained from metabolomic and transcriptomic analyses were overlapped. For pathways significantly enriched in both datasets, correlation networks between differentially expressed genes and differential metabolites were constructed based on Spearman correlation coefficients (|r| > 0.6) and visualized using Cytoscape software 3.10.4.

### 2.9. Antioxidant Enzyme Activity Assays

Liver tissues were homogenized in ice-cold phosphate-buffered saline (PBS) and centrifuged at 8000× *g* for 10 min at 4 °C. The resulting supernatants were used to determine the activities of superoxide dismutase (SOD), catalase (CAT), and glutathione peroxidase (GPX).

SOD activity was measured using the xanthine–xanthine oxidase method based on inhibition of nitroblue tetrazolium reduction. CAT activity was determined using the ammonium molybdate method by measuring residual hydrogen peroxide (H_2_O_2_). GPX activity was assayed by monitoring the consumption of reduced glutathione (GSH) in the presence of H_2_O_2_. All enzyme activities were measured using a microplate reader (SpectraMax ABS Plus, Molecular Devices, San Jose, CA, USA). Protein concentration was determined using the bicinchoninic acid (BCA) method.

### 2.10. Statistical Analysis

All data are presented as mean ± standard deviation (SD). Statistical analyses were performed using GraphPad Prism 9.0 (GraphPad Software, San Diego, CA, USA). Differences among experimental groups were analyzed using Student’s *t*-test. A value of *p* < 0.05 was considered statistically significant.

## 3. Results

### 3.1. Dietary HMS Supplementation Improves Survival Without Affecting Growth Performance

**Figure 1:**
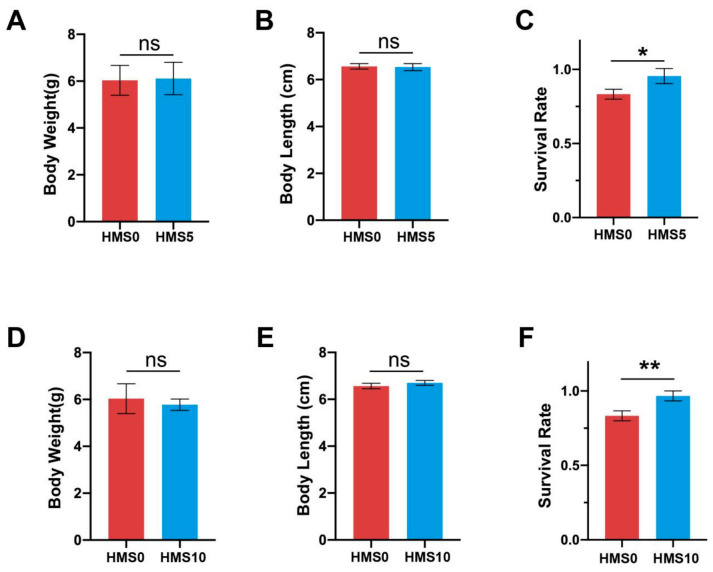
Effects of dietary H. marmoreus stipe (HMS) supplementation on growth performance and survival rate of largemouth bass. (**A**–**C**) Comparison of body weight (**A**), body length (**B**), and survival rate (**C**) between HMS5 (5% HMS) and HMS0 (0% HMS) groups. (**D**–**F**) Comparison of body weight (**D**), body length (**E**), and survival rate (**F**) between HMS10 (10% HMS) and HMS0 groups. Data are presented as mean ± SD (*n* = 3 replicate tanks per group). Statistical significance was determined by Student’s *t*-test, * *p* < 0.05, ** *p* < 0.01, ns means no significant difference between two groups.

After 46 days of feeding, no significant differences in final body weight or body length were observed between the HMS5 and HMS0 groups (Figure 1A,B). Similarly, fish fed the HMS10 diet showed no significant changes in body weight or body length compared with the HMS0 group (Figure 1D,E). Consistent with this finding, the FCR and SGR of fish exhibited no remarkable differences across all experimental groups (Table 2). Only the condition factor displayed slight statistical discrepancies among treatments (Table 2). However, the survival rates of fish in the HMS0, HMS5 and HMS10 groups were 83.33%, 95.56% and 96.67%, respectively. Compared with the HMS0 group, significantly improved survival was detected in the HMS5 (*p* < 0.05) and HMS10 (*p* < 0.01) groups (Figure 1C,F). Fish mortality occurred progressively without acute mass mortality. Furthermore, no apparent macroscopic pathological lesions or characteristic clinical signs of infectious diseases were identified in any dead individuals. These results indicate that dietary HMS supplementation improved the survival of juvenile *Micropterus salmoides* without significantly affecting growth performance.

### 3.2. Dietary HMS Supplementation Alters Liver and Stomach Metabolic Profiles

**Figure 2:**
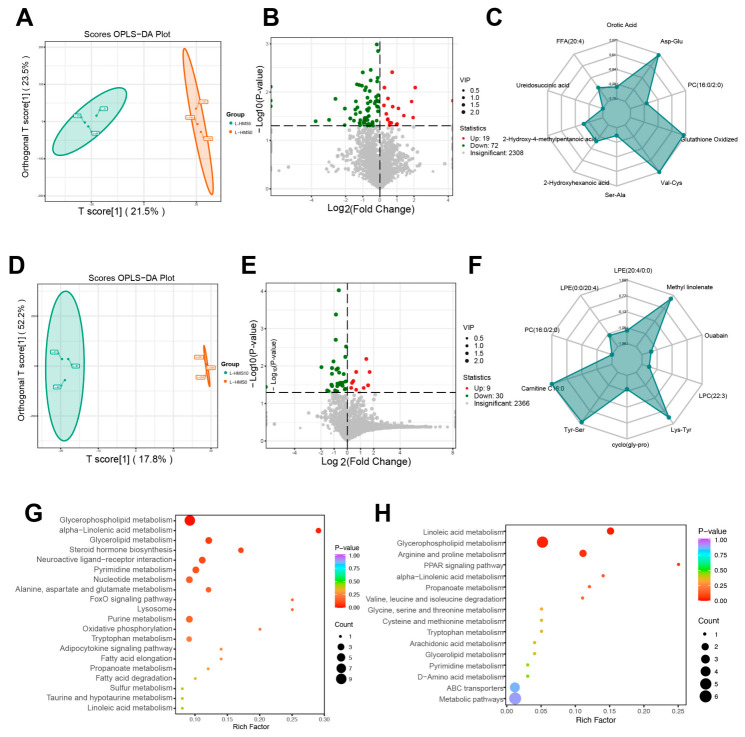
Liver metabolomic profiles of largemouth bass fed HMS-supplemented diets. (**A**) OPLS-DA score plot of HMS5 vs. HMS0 in liver samples, R^2^X = 0.451, R^2^Y = 0.995, Q^2^ = 0.463. (**B**) Volcano plot of differential metabolites in HMS5 vs. HMS0 (VIP > 1, *p* < 0.05). (**C**) Radar plot of the top 10 differential metabolites with highest |log_2_FC| in HMS5 vs. HMS0. (**D**) OPLS-DA score plot of HMS10 vs. HMS0 in liver samples, R^2^X = 0.829, R^2^Y = 0.999, Q^2^ = 0.638. (**E**) Volcano plot of differential metabolites in HMS10 vs. HMS0. (**F**) Radar plot of the top 10 differential metabolites in HMS10 vs. HMS0. (**G**) KEGG pathway enrichment analysis of differential metabolites in HMS5 vs. HMS0. (**H**) KEGG pathway enrichment analysis of differential metabolites in HMS10 vs. HMS0.

**Figure 3:**
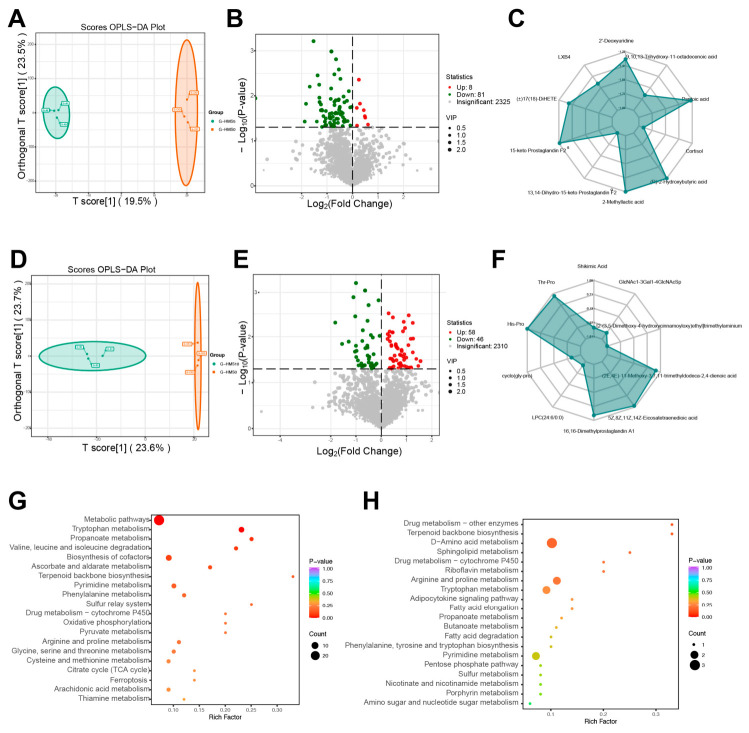
Stomach metabolomic profiles of largemouth bass fed HMS-supplemented diets. (**A**) OPLS-DA score plot of HMS5 vs. HMS0 in stomach samples, R^2^X = 0.430, R^2^Y = 0.999, Q^2^ = 0.539. (**B**) Volcano plot of differential metabolites in HMS5 vs. HMS0 (VIP > 1, *p* < 0.05). (**C**) Radar plot of the top 10 differential metabolites in HMS5 vs. HMS0. (**D**) OPLS-DA score plot of HMS10 vs. HMS0 in stomach samples, R^2^X = 0.473, R^2^Y = 0.991, Q^2^ = 0.303. (**E**) Volcano plot of differential metabolites in HMS10 vs. HMS0. (**F**) Radar plot of the top 10 differential metabolites in HMS10 vs. HMS0. (**G**) KEGG pathway enrichment analysis of differential metabolites in HMS5 vs. HMS0. (**H**) KEGG pathway enrichment analysis of differential metabolites in HMS10 vs. HMS0.

Widely targeted metabolomic profiling identified extensive metabolic alterations in both liver and stomach tissues following dietary HMS supplementation. Orthogonal partial least squares-discriminant analysis (OPLS-DA) revealed clear separation between the HMS5 and HMS0 groups in both liver (Figure 2A) and stomach tissues (Figure 3A), as well as between the HMS10 and HMS0 groups (Figure 2D and Figure 3D), indicating distinct metabolic profiles among treatments.

In liver tissues, comparison between HMS5 and HMS0 identified 19 up-regulated and 72 down-regulated metabolites (Figure 2B), whereas HMS10 vs. HMS0 identified 9 up-regulated and 30 down-regulated metabolites (Figure 2E). The top 10 differential metabolites ranked by absolute log2 fold change are presented in Figure 2C,F. KEGG enrichment analysis showed that differential metabolites in the HMS5 group were primarily enriched in glycerophospholipid metabolism, steroid hormone biosynthesis, pyrimidine metabolism, and tryptophan metabolism (Figure 2G). In the HMS10 group, significantly enriched pathways included glycerophospholipid metabolism, tryptophan metabolism, pyrimidine metabolism, and ABC transporters (Figure 2H).

In stomach tissues, HMS5 vs. HMS0 showed 8 up-regulated and 81 down-regulated metabolites (Figure 3B), whereas HMS10 vs. HMS0 exhibited 58 up-regulated and 46 down-regulated metabolites (Figure 3E). KEGG enrichment analysis revealed that differential metabolites in the HMS5 group were mainly associated with tryptophan metabolism and pyrimidine metabolism, while those in the HMS10 group were enriched in pyrimidine metabolism and sphingolipid metabolism (Figure 3G,H).

### 3.3. Hepatic Transcriptomic Profiling Reveals HMS-Regulated Gene Expression Patterns

**Figure 4:**
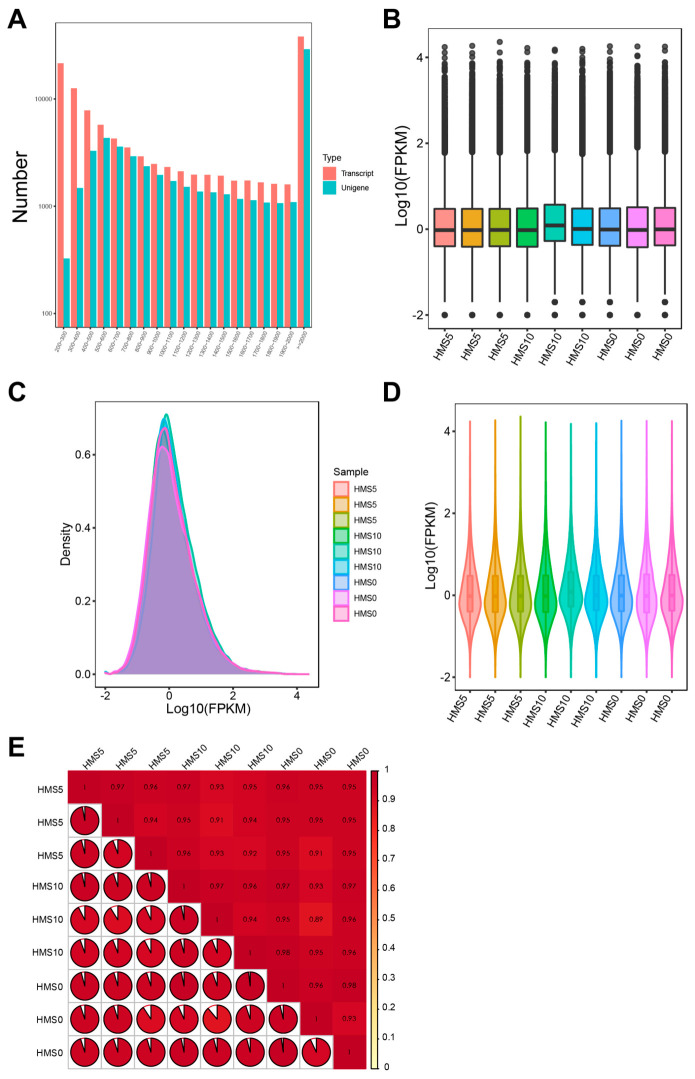
Quality assessment of de novo liver transcriptome assembly and gene expression profiling. (**A**) Sequence length distribution of assembled transcripts. The majority of transcripts ranged from 500 to 2000 bp, indicating a high-quality assembly. (**B**) Boxplot of log10(FPKM) values across samples. The median and interquartile ranges were comparable among all groups, suggesting similar global expression patterns. (**C**) Density plot of gene expression levels. The curves overlapped well across samples, indicating consistent expression distributions. (**D**) Violin plot showing the probability density of expression levels. The shape and width of the violins were similar among replicates, confirming data homogeneity. (**E**) Sample correlation heatmap based on Pearson‘s correlation coefficients. All biological replicates showed r > 0.9, demonstrating excellent reproducibility.

De novo transcriptome assembly generated 62,133 unigenes, with an N50 length of 3851 bp and an average transcript length of 2480 bp. BUSCO analysis demonstrated high assembly completeness, indicating reliable transcriptome quality. Gene expression distributions among all samples showed good consistency and acceptable dispersion, as demonstrated by boxplot, density plot, and violin plot analyses (Figure 4B–D). In addition, Pearson correlation coefficients among biological replicates exceeded 0.9, indicating high reproducibility of the transcriptomic datasets (Figure 4E).

**Figure 5:**
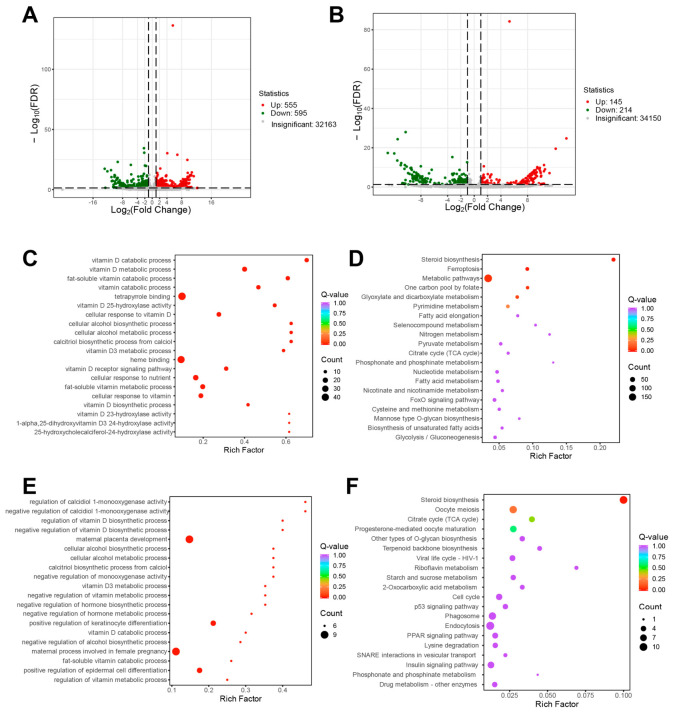
Differential gene expression and functional enrichment in liver of HMS-fed largemouth bass. (**A**) Volcano plot of differentially expressed genes (DEGs) in HMS5 vs. HMS0 (|log_2_FC| ≥ 1, FDR < 0.05). The two vertical dashed lines correspond to the threshold of |log_2_FC| = 1. (**B**) Volcano plot of DEGs in HMS10 vs. HMS0. (**C**) GO enrichment analysis of DEGs in HMS5 vs. HMS0. (**D**) KEGG pathway enrichment analysis of DEGs in HMS5 vs. HMS0. (**E**) GO enrichment analysis of DEGs in HMS10 vs. HMS0. (**F**) KEGG pathway enrichment analysis of DEGs in HMS10 vs. HMS0.

Differential expression analysis identified 555 up-regulated and 595 down-regulated genes in the HMS5 vs. HMS0 comparison, whereas 145 up-regulated and 214 down-regulated genes were identified in HMS10 vs. HMS0 (Figure 5A,B). GO enrichment analysis of up-regulated genes in the HMS5 group revealed enrichment in terms related to vitamin D metabolic process, tetrapyrrole binding, and vitamin D3 metabolic process (Figure 5C). KEGG pathway analysis showed significant enrichment of pyrimidine metabolism and pyruvate metabolism pathways (Figure 5D). In the HMS10 group, enriched GO terms included vitamin D3 metabolic process (Figure 5E), while KEGG pathways included steroid biosynthesis and PPAR signaling pathway (Figure 5F).

### 3.4. Integrated Multi-Omics Analysis Identifies Key Pathways Associated with the Effects of HMS Supplementation

**Figure 6:**
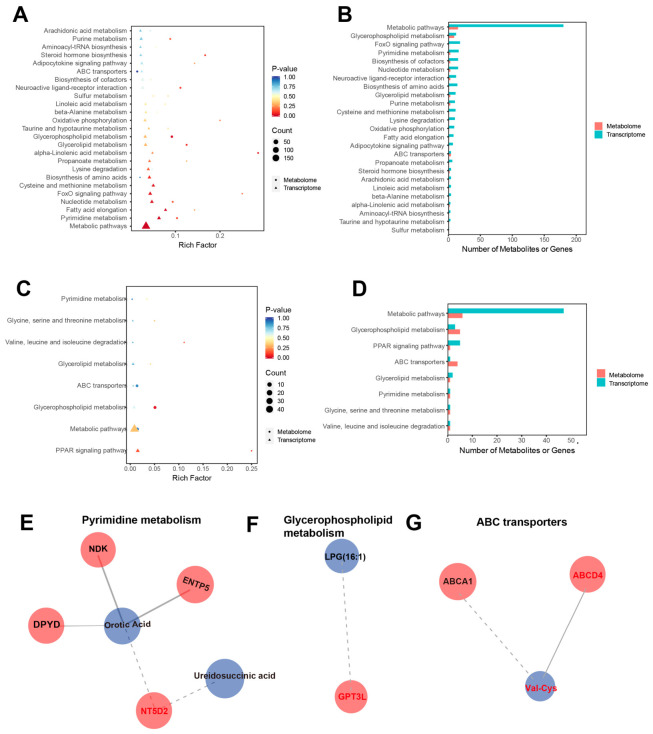
Integrated metabolomic and transcriptomic analysis of liver. (**A**) Bubble plot and (**B**) bar plot showing overlapping KEGG pathways enriched by both differential metabolites and DEGs in HMS5 vs. HMS0. (**C**) Bubble plot and (**D**) bar plot showing overlapping KEGG pathways in HMS10 vs. HMS0. Three pathways (glycerophospholipid metabolism, pyrimidine metabolism, and ABC transporters) were commonly enriched in both comparisons. (**E**) Correlation network of pyrimidine metabolism: DPYD, NDK, ENTP5, and NT5D2 (genes) correlated with orotic acid and ureidosuccinic acid (metabolites). (**F**) Correlation network of glycerophospholipid metabolism: GPT3L correlated with LPG (16:1). (**G**) Correlation network of ABC transporters: ABCA1 and ABCD4 correlated with the dipeptide Val-Cys. Red nodes indicate up-regulated molecules, black nodes indicate down-regulated molecules. Solid lines represent positive correlations, and dashed lines represent negative correlations.

To investigate the molecular pathways associated with the beneficial effects of dietary HMS supplementation, integrated analyses of hepatic metabolomic and transcriptomic datasets were performed. Overlap analysis of KEGG enrichment results revealed three pathways that were commonly enriched in both HMS5 vs. HMS0 and HMS10 vs. HMS0 comparisons: pyrimidine metabolism, glycerophospholipid metabolism, and ABC transporters (Figure 6A–D). These pathways showed coordinated alterations at both metabolite and transcript levels.

Correlation network analysis further revealed potential associations between differentially expressed genes and metabolites within these pathways. In pyrimidine metabolism, four genes—*DPYD* (dihydropyrimidine dehydrogenase), *NDK* (nucleoside diphosphate kinase), *ENTP5* (ectonucleoside triphosphate diphosphohydrolase 5), *NT5D2* (5′-nucleotidase domain containing 2)—showed strong correlations with the metabolites orotic acid and ureidosuccinic acid (Figure 6E). Within glycerophospholipid metabolism, GPT3L (glycerol-3-phosphate acyltransferase-like) exhibited a strong negative correlation with lysophospholipid LPG (16:1) (Figure 6F). In the ABC transporter pathway, ABCD4 (ATP-binding cassette subfamily D member 4) showed a positive correlation with the dipeptide Val-Cys, whereas ABCA1 (ATP-binding cassette subfamily A member 1) displayed a negative correlation with Val-Cys (Figure 6G).

Collectively, these results suggest that dietary HMS supplementation may induced coordinated changes in pyrimidine metabolism, glycerophospholipid metabolism, and ABC transporter-related pathways.

### 3.5. Dietary HMS Supplementation Modulates Antioxidant Enzyme Activities

**Figure 7:**
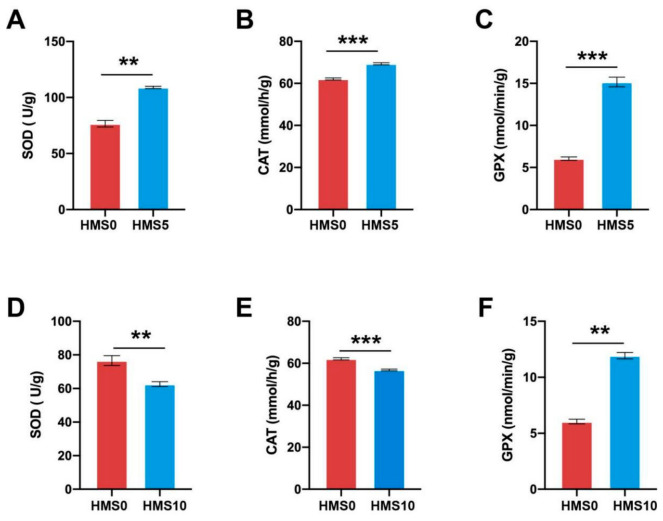
Effects of HMS supplementation on hepatic antioxidant enzyme activities. (**A**–**C**) Activities of superoxide dismutase (SOD) (**A**), catalase (CAT) (**B**), and glutathione peroxidase (GPX) (**C**) in HMS5 vs. HMS0. (**D**–**F**) Activities of SOD (**D**), CAT (**E**), and GPX (**F**) in HMS10 vs. HMS0. HMS5 significantly increased SOD, CAT, and GPX activities compared to HMS0. In contrast, HMS10 increased GPX activity but decreased SOD and CAT activities. Data are presented as mean ± SD (*n* = 3). ** *p* < 0.01, *** *p* < 0.001.

Compared with the HMS0 group, fish fed the HMS5 diet exhibited significantly increased activities of superoxide dismutase (SOD), catalase (CAT), and glutathione peroxidase (GPX) (*p* < 0.01 or *p* < 0.001; Figure 7A–C). In contrast, the HMS10 group showed reduced SOD and CAT activities compared with the HMS0 group, whereas GPX activity remained elevated (Figure 7D–F). These results indicate that dietary HMS supplementation exerted dose-dependent effects on antioxidant enzyme activities in juvenile largemouth bass.

## 4. Discussion

This study demonstrated for the first time that dietary supplementation with Hypsizygus marmoreus stipe (HMS) at 5% and 10% significantly improved the survival rate of juvenile *Micropterus salmoides* without affecting growth performance. Integrated metabolomic and transcriptomic analyses further revealed that HMS supplementation coordinately regulated pyrimidine metabolism, glycerophospholipid metabolism, and ABC transporter pathways. This tends to suggest that these pathways are closely associated with the survival-promoting effects of HMS.

Both metabolomic and transcriptomic analyses consistently identified pyrimidine metabolism as a key pathway affected by HMS supplementation. In the integrated correlation network, orotic acid and ureidosuccinic acid—two core intermediates of the de novo pyrimidine synthesis pathway—were significantly downregulated in the liver of HMS-treated largemouth bass. Concurrently, transcript levels of catabolism-related genes including *DPYD*, *NDK*, and *ENTP5* were suppressed, while *NT5D2*, encoding a rate-limiting enzyme of the nucleotide salvage pathway, was significantly upregulated. Two mutually plausible mechanistic interpretations may account for the reduced abundance of orotic acid and ureidosuccinic acid, and the present multi-omics dataset lacks direct metabolic flux measurements to fully discriminate between these two scenarios.

On one hand, the lowered intermediate concentrations may reflect accelerated downstream consumption of newly generated pyrimidines. Under this framework, three coordinated metabolic shifts jointly drive nucleotide conservation and recycling: (1) repressed pyrimidine breakdown via downregulation of DPYD, NDK, and ENTP5, which limits the degradation of endogenous nucleotide reserves; (2) boosted salvage pathway capacity mediated by elevated NT5D2 expression, enabling efficient recycling of free purine and pyrimidine bases; (3) accelerated turnover of pyrimidine intermediates to support DNA and RNA biogenesis during immune cell proliferation and hepatic tissue repair, which would rapidly deplete orotic acid and ureidosuccinic acid pools. On the other hand, the diminished levels of these two intermediates could equally arise from reduced metabolic flux through the de novo pyrimidine synthesis pathway itself, which would also lead to lower steady-state concentrations of upstream pathway metabolites. Collectively, the transcriptional and metabolic profiles point to a global metabolic reprogramming oriented toward nucleotide preservation and recycling, though definitive discrimination between enhanced downstream utilization and attenuated de novo synthetic flux requires further studies with larger sample sizes.

Pyrimidine nucleotides are essential not only for nucleic acid synthesis but also for cellular energy metabolism, signal transduction, and the biosynthesis of glycoproteins and phospholipids. In fish, adequate nucleotide availability is critical for immune function because lymphocytes and macrophages require rapid nucleotide turnover during activation and clonal expansion. Several studies have demonstrated that dietary nucleotide supplementation enhances disease resistance and survival in fish. For example, Chen et al. reported that dietary nucleotide supplementation improved growth performance, immune responses, and resistance against *Aeromonas hydrophila* infection in juvenile largemouth bass [13]. Similarly, dietary nucleotides suppressed intestinal inflammation and enhanced disease resistance in grass carp (*Ctenopharyngodon idella*) through NF-κB and TOR signaling pathways [18]. The physiological significance of pyrimidine metabolic regulation in stress adaptation is further supported by Ali et al., who demonstrated that a pyrimidine analog improved hematological parameters and moderated oxidative stress in freshwater stinging catfish (*Heteropneustes fossilis*) without affecting growth performance [19]. These findings are consistent with our observation that HMS improved survival without significantly altering growth. Collectively, our results suggest that HMS supplementation does not merely stimulate de novo pyrimidine synthesis but rather promotes a metabolic state favoring nucleotide conservation and efficient recycling. Such metabolic reprogramming may help maintain nucleotide homeostasis under culture-related stress conditions, thereby supporting immune function and improving fish survival.

Integrated metabolomic and transcriptomic analyses also revealed significant enrichment of glycerophospholipid metabolism in HMS-supplemented groups. In the correlation network, GPT3L (glycerol-3-phosphate acyltransferase-like) was up-regulated, whereas LPG (16:1) (lysophosphatidylglycerol) was down-regulated, with a strong negative correlation between them. These results suggest that HMS supplementation promoted membrane phospholipid biosynthesis while reducing lysophospholipid accumulation. Glycerophospholipids are essential structural components of cellular membranes and also serve as precursors for immune-related signaling molecules such as prostaglandins and platelet-activating factor [20,21,22,23,24,25]. Therefore, up-regulation of GPT3L may enhance membrane remodeling capacity and lipid mediator synthesis, both of which are important for stress adaptation and immune responses. In contrast, LPG (16:1) is a lysophospholipid generated through phospholipase A2-mediated phospholipid hydrolysis [26]. Elevated lysophospholipid levels are frequently associated with membrane damage and inflammatory responses in fish [27]. Thus, the reduction in LPG (16:1) observed in the present study may reflect improved membrane stability and reduced inflammatory stress.

The negative correlation between GPT3L and LPG (16:1) is biologically plausible because enhanced phospholipid biosynthesis may reduce phospholipid degradation and subsequent lysophospholipid accumulation. This coordinated metabolic shift appears to favor membrane maintenance over membrane breakdown. Previous studies have also highlighted the importance of glycerophospholipid metabolism in fish health. In mandarin fish, this pathway is associated with immune regulation and feed adaptation, whereas disruption of glycerophospholipid metabolism induces oxidative stress and immunotoxicity in turbot [28,29]. In largemouth bass, dietary lysophospholipid supplementation has been reported to improve hepatic lipid metabolism [30,31,32]. Together, these findings may support the hypothesis that HMS enhances membrane integrity and alleviates excessive inflammatory responses through modulation of glycerophospholipid metabolism, thereby contributing to improved survival.

ABC transporters emerged as another important pathway identified through integrated analysis. Within the correlation network, ABCD4 (ATP-binding cassette subfamily D member 4) was up-regulated and positively correlated with the dipeptide Val-Cys, whereas ABCA1 was down-regulated and negatively correlated with Val-Cys. These results indicate differential regulation of ABC transporter family members in response to HMS supplementation. ABCD4 is localized to lysosomal membranes and mediates transport of vitamin B12 from lysosomes into the cytosol [33]. Vitamin B12 functions as an essential cofactor for methionine synthase, which links methionine metabolism to glutathione (GSH) biosynthesis [34,35]. Therefore, up-regulation of ABCD4 may facilitate GSH synthesis by improving intracellular vitamin B12 availability. Val-Cys is a cysteine-containing dipeptide, and cysteine is the rate-limiting substrate for GSH synthesis. Previous studies have shown that cysteine-containing peptides possess antioxidant properties and can effectively scavenge reactive oxygen species [36]. Accordingly, the positive correlation between ABCD4 and Val-Cys suggests that HMS supplementation may enhance antioxidant capacity through coordinated regulation of vitamin B12 transport and cysteine availability.

ABCA1 is a well-characterized plasma membrane transporter involved in cholesterol efflux and lipid homeostasis [37]. In fish, ABCA1 participates in hepatic cholesterol and lipid transport, and reduced ABCA1 expression has been associated with metabolic fatty liver disease in largemouth bass [38]. In addition, ABCA1 deficiency in macrophages is associated with elevated pro-inflammatory gene expression and cytokine release [37]. The reduced expression of ABCA1 observed in the present study may therefore represent an adaptive response associated with altered lipid metabolism and inflammatory regulation. Furthermore, the negative correlation between ABCA1 and Val-Cys suggests coordinated regulation between lipid transport and antioxidant metabolism. Taken together, these findings indicate that HMS supplementation may improve cellular protection by simultaneously enhancing antioxidant capacity and modulating lipid-associated inflammatory responses.

An important finding of the present study was the dose-dependent biphasic response of hepatic antioxidant enzyme activities induced by graded HMS inclusion. Specifically, dietary supplementation with 5% HMS significantly elevated hepatic SOD, CAT, and GPX activities compared with the HMS0 control, whereas 10% HMS markedly suppressed hepatic SOD and CAT activities to levels even lower than the blank group, despite retaining relatively higher GPX activity. This distinct phenotypic divergence between low and high inclusion levels conforms to a typical hormetic response, in which moderate doses of fungal bioactive compounds trigger adaptive protective antioxidant cascades, whereas excessive supplementation disrupts hepatic redox homeostasis and elicits direct inhibitory impacts on core antioxidant enzymes [39,40]. The raw HMS substrate contains approximately 20% crude fiber; excessive insoluble fiber within the digestive tract physically impedes the absorption of antioxidant precursors and essential nutrients, disturbs intestinal bile acid circulation and microbiota balance, and ultimately aggravates endogenous reactive oxygen species accumulation to overwhelm the hepatic antioxidant defense system. Meanwhile, mushroom-derived bioactive compounds, including polysaccharides, free amino acids and phenolic substances, are widely documented to exert concentration-dependent dual antioxidant or pro-oxidant effects [41]. At the 5% inclusion level, the beneficial bioactive components predominate and counteract mild oxidative stress, while the adverse interference from high crude fiber becomes dominant at the 10% threshold, offsetting the protective effects of mushroom extracts and depressing SOD and CAT expression. Combined with the prominently improved survival rate recorded in the HMS5 group, these integrated physiological and survival outcomes collectively indicate that 5% HMS appeared to be the most favorable level among the tested doses for juvenile largemouth bass under the present experimental conditions to balance growth, survival and hepatic antioxidant capacity. Comparable dose-dependent biphasic hormetic effects triggered by mushroom-based feed additives have also been well documented in multiple previous aquaculture investigations [16].

Several limitations of this study should be acknowledged. First, the experimental diets replaced multiple basal ingredients (fish meal, flour, fermented soybean meal, brewer yeast, and fish oil) with HMS simultaneously. Accordingly, the physiological responses observed may stem from overall variations in feed composition rather than solely bioactive substances derived from HMS. Second, pathogen challenge experiments were not conducted; therefore, the observed increase in survival may primarily reflect improved general physiological resilience rather than enhanced pathogen-specific immunity. Third, the specific bioactive compounds responsible for the beneficial effects of HMS remain unclear. Future studies should isolate and characterize individual components, such as polysaccharides, ergosterol, and phenolic compounds, to determine their respective biological activities. Fourth, only liver and stomach tissues were analyzed in this study. Considering the relatively high crude fiber content of HMS, future investigations focusing on gut microbiota and intestinal health may provide additional mechanistic insights. Finally, longer-term feeding trials with more refined supplementation gradients are required to determine the optimal dietary inclusion level for practical aquaculture applications.

## 5. Conclusions

This study demonstrated that dietary supplementation with *H. marmoreus* stipe (HMS) significantly improved the survival rate of juvenile *M. salmoides* without affecting growth performance, with 5% supplementation showing the most pronounced beneficial effect. Integrated widely targeted metabolomic and de novo transcriptomic analyses suggested that HMS supplementation may coordinately modulate three major metabolic pathways in the liver, including pyrimidine metabolism, glycerophospholipid metabolism, and ABC transporter-related pathways. Specifically, HMS supplementation was associated with suppressed pyrimidine catabolism and enhanced nucleotide salvage activity, increased glycerophospholipid biosynthesis accompanied by reduced lysophospholipid accumulation, and altered ABC transporter regulation linked to antioxidant metabolism. These coordinated metabolic changes may contribute to improved nucleotide homeostasis, membrane integrity, and antioxidant capacity, thereby enhancing fish survival under culture conditions. In addition, antioxidant enzyme activities exhibited a dose-dependent biphasic response, suggesting that moderate HMS supplementation exerts more favorable physiological effects than excessive supplementation. Overall, our findings support the potential application of mushroom-processing byproducts as sustainable functional feed ingredients in aquaculture and provide new insights into the metabolic mechanisms underlying nutritional regulation of fish survival.

## Figures and Tables

**Table 1:** Composition and proximate analysis of the experimental diets.

Ingredient (g/100 g)	HMS0	HMS5	HMS10
Fish meal	40.00	37.50	35.79
HMS	0.00	4.84	9.23
Flour	22.00	21.28	20.30
Fermented soybean meal	15.00	14.51	13.84
Brewer yeast	5.00	4.84	4.61
Corn protein meal	5.00	4.84	4.61
Vitamin premix	1.00	0.97	0.92
Mineral salt premix	1.00	0.97	0.92
Calcium dihydrogen phosphate	1.20	1.16	1.11
Fish oil	4.00	3.48	3.32
Choline chloride	0.60	0.58	0.55
Microcrystalline micronutrient	5.20	5.03	4.80
Proximate analysis	-	-	-
Crude protein	44.73	44.73	44.73
Crude lipid	7.87	7.87	7.87
Crude fibre	1.20	2.17	3.05
Ash	8.50	9.08	9.60

Note: Each kilogram of vitamin premix contains: vitamin A 80,000 IU, vitamin D_3_ 300,000 IU, vitamin K 800 mg, vitamin B_1_ 1500 mg, vitamin B_2_ 2000 mg, vitamin B_6_ 1600 mg, vitamin B_12_ 50 mg, vitamin C 20 g, pantothenic acid 3200 mg, folic acid 500 mg and inositol 30 g; per kilogram of mineral salt premix contains: KCl 60 g, NaCl 40 g, FeCl 40 g, ZnSO_4_ 30 g, MeSO_4_ 30 g, and CoSO_4_ 2 g.

**Table 2:** Growth performance and feed utilization parameters of the experimental diets.

Group	HMS0	HMS5	HMS10
FCR	0.62 ± 0.08 ^a^	0.67 ± 0.09 ^a^	0.72 ± 0.02 ^a^
SGR (%/day)	2.71 ± 0.18 ^a^	2.45 ± 0.17 ^a^	2.55 ± 0.08 ^a^
CF (g/cm^3^)	0.02 ± 0.00 ^ab^	0.02 ± 0.00 ^a^	0.02 ± 0.00 ^b^

Note: Values are presented as mean ± SE. Different lowercase letters within the same column indicate significant differences among the three treatments (*p* < 0.05), whereas values sharing the same lowercase letter are not significantly different (*p* > 0.05).

## Data Availability

The original contributions presented in this study are included in the article. Further inquiries can be directed to the corresponding author.
